# Proteo-Genomic Analysis Identifies Two Major Sites of Vulnerability on Ebolavirus Glycoprotein for Neutralizing Antibodies in Convalescent Human Plasma

**DOI:** 10.3389/fimmu.2021.706757

**Published:** 2021-07-16

**Authors:** Pavlo Gilchuk, Adrian Guthals, Stefano R. Bonissone, Jared B. Shaw, Philipp A. Ilinykh, Kai Huang, Robin G. Bombardi, Jenny Liang, Ariadna Grinyo, Edgar Davidson, Elaine C. Chen, Bronwyn M. Gunn, Galit Alter, Erica Ollmann Saphire, Benjamin J. Doranz, Alexander Bukreyev, Larry Zeitlin, Natalie Castellana, James E. Crowe

**Affiliations:** ^1^ The Vanderbilt Vaccine Center, Vanderbilt University Medical Center, Nashville, TN, United States; ^2^ Mapp Biopharmaceutical, Inc. San Diego, CA, United States; ^3^ Abterra Biosciences (formerly Digital Proteomics LLC), San Diego, CA, United States; ^4^ Environmental Molecular Sciences Laboratory, Pacific Northwest National Laboratory, Richland, WA, United States; ^5^ Department of Pathology, University of Texas Medical Branch, Galveston, TX, United States; ^6^ Galveston National Laboratory, Galveston, TX, United States; ^7^ Integral Molecular, Inc., Philadelphia, PA, United States; ^8^ Department of Pathology, Microbiology, and Immunology, Vanderbilt University Medical Center, Nashville, TN, United States; ^9^ Ragon Institute of MGH, MIT, and Harvard, Cambridge, MA, United States; ^10^ Center for Infectious Disease and Vaccine Research, La Jolla Institute for Immunology, La Jolla, CA, United States; ^11^ Department of Microbiology & Immunology, University of Texas Medical Branch, Galveston, TX, United States; ^12^ Department of Pediatrics, Vanderbilt University Medical Center, Nashville, TN, United States

**Keywords:** ebolavirus, ebolavirus infection, glycoprotein, proteo-genomics, convalescent plasma, viral antibodies, neutralizing antibodies, epitope mapping

## Abstract

Three clinically relevant ebolaviruses – Ebola (EBOV), Bundibugyo (BDBV), and Sudan (SUDV) viruses, are responsible for severe disease and occasional deadly outbreaks in Africa. The largest Ebola virus disease (EVD) epidemic to date in 2013-2016 in West Africa highlighted the urgent need for countermeasures, leading to the development and FDA approval of the Ebola virus vaccine rVSV-ZEBOV (Ervebo^®^) in 2020 and two monoclonal antibody (mAb)-based therapeutics (Inmazeb^®^ [atoltivimab, maftivimab, and odesivimab-ebgn] and Ebanga^®^ (ansuvimab-zykl) in 2020. The humoral response plays an indispensable role in ebolavirus immunity, based on studies of mAbs isolated from the antibody genes in peripheral blood circulating ebolavirus-specific human memory B cells. However, antibodies in the body are not secreted by circulating memory B cells in the blood but rather principally by plasma cells in the bone marrow. Little is known about the protective polyclonal antibody responses in convalescent plasma. Here we exploited both single-cell antibody gene sequencing and proteomic sequencing approaches to assess the composition of the ebolavirus glycoprotein (GP)-reactive antibody repertoire in the plasma of an EVD survivor. We first identified 1,512 GP-specific mAb variable gene sequences from single cells in the memory B cell compartment. Using mass spectrometric analysis of the corresponding GP-specific plasma IgG, we found that only a portion of the large B cell antibody repertoire was represented in the plasma. Molecular and functional analysis of proteomics-identified mAbs revealed recognition of epitopes in three major antigenic sites - the GP head domain, the glycan cap, and the base region, with a high prevalence of neutralizing and protective mAb specificities that targeted the base and glycan cap regions on the GP. Polyclonal plasma antibodies from the survivor reacted broadly to EBOV, BDBV, and SUDV GP, while reactivity of the potently neutralizing mAbs we identified was limited mostly to the homologous EBOV GP. Together these results reveal a restricted diversity of neutralizing humoral response in which mAbs targeting two antigenic sites on GP – glycan cap and base – play a principal role in plasma-antibody-mediated protective immunity against EVD.

## Introduction

Ebolaviruses are responsible for severe disease and occasional deadly outbreaks in Africa posing a significant health threat. The Ebolavirus genus consists of six species, including Zaire ebolavirus [represented by Ebola virus (EBOV)], Sudan ebolavirus [Sudan virus (SUDV)], Bundibugyo ebolavirus [Bundibugyo virus (BDBV)], Taï Forest ebolavirus [Taï Forest virus (TAFV)], Reston ebolavirus [Reston virus (RESV)] (1), and Bombali ebolavirus [Bombali virus (BOMV)] (2). EBOV, BDBV, and SUDV are the medically important causative agents of symptomatic infections and ebolavirus disease (EVD) in humans. A total of 41 confirmed EVD outbreaks have been documented, and the largest EVD epidemic to date occurred in 2013-2016 in West Africa with a total of 28,610 disease cases and 11,308 deaths reported (3). The unpredictable nature of EVD outbreaks and public health challenges stemming from the severity of the disease underscores the need for development of medical countermeasures and systematic studies to elucidate correlates of immune response-mediated protection against EVD.

The evidence to date suggests an indispensable role for antibody-mediated immunity in the protection against EVD. Several investigational treatments based on human monoclonal antibodies (mAb) showed therapeutic efficacy in animal models of EVD (4–8) and clinical trials in the Democratic Republic of Congo outbreak demonstrated high efficacy of antibody-based therapeutics for acute EVD treatment in patients (9). By 2020, two monoclonal antibody-based therapeutics – ansuvimab-zykl (Ebanga^®^) and atoltivimab + maftivimab + odesivimab-ebgn (Inmazeb^®^) – were developed and approved by the Food and Drug Administration (FDA) for clinical use (10, 11). A landmark achievement was the development and FDA approval of a recombinant viral vector-based vaccine (Ervebo^®^) for prevention of EVD (12, 13), vaccination with which has been shown to induce long-lasting antibody responses in clinical trials (14).

The key target for protective antibodies is the ebolavirus glycoprotein (GP), which is a single surface protein of the viral envelope. GP forms a trimer, in which each protomer consists of two subunits, designated GP1 and GP2. The GP1 subunit contains a heavily glycosylated mucin-like domain (MLD) and a glycan cap, which shields the host receptor binding site (RBS). The RBS is exposed after proteolytical cleavage in the host endosome and binds to domain C of its endosomal receptor, the protein Niemann-Pick C1 (NPC1-C). The GP2 subunit contains the internal fusion loop (IFL) and stalk and is anchored into the viral membrane by a transmembrane domain (15–17).

Recent improvement of instruments and technologies for high-throughput single B cell analysis enabled isolation of thousands of ebolavirus GP-reactive mAbs from the circulating memory B cells of EVD survivors or vaccinees (18–20). Hundreds of mAbs were characterized at the molecular level in studies that revealed a diverse landscape of epitope recognition in which distinct classes of mAbs recognized the MLD, glycan cap, GP1 head, GP1/GP2 trimer base, IFL, or stalk regions on the GP (21). This and other studies also demonstrated that only a fraction of ebolavirus GP-reactive mAbs protects against infection or disease *in vivo* (18, 22, 23). Nevertheless, protection is mediated principally by antibodies circulating in serum that are secreted by long-lived plasma cells in the bone marrow, not by circulating memory B cells (24). Bulk serological analysis of plasma from four EVD survivors suggested preferential binding of polyclonal plasma antibodies to proteolytically-cleaved form of GP (18). The landscape of epitope recognition by protective polyclonal antibody responses and their prevalence in convalescent plasma remains unknown.

**Figure 1:**
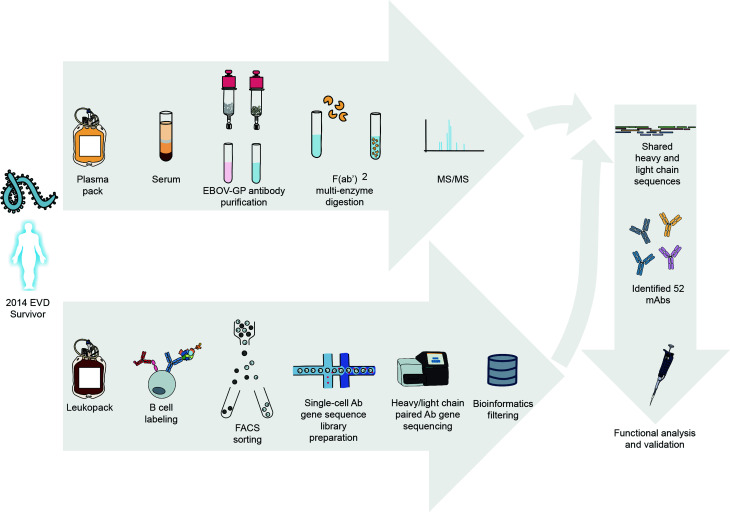
Proteo-genomics workflow for the discovery of ebolavirus GP-specific monoclonal antibodies in convalescent human plasma. A cartoon representation is shown of key workflow steps, which included collection of convalescent plasma from EVD survivor, affinity purification of polyclonal GP-specific antibodies from plasma, mass-spectrometric analysis of plasma antibody protein sequences, isolation of GP-specific B cells from the peripheral blood mononuclear cells of an EVD survivor, single-cell V(D)J gene sequence analysis, bioinformatics analysis to identify shared proteo-genomic antibody heavy and light chain variable region sequences, recombinant expression of identified monoclonal antibodies, and functional analysis of recombinant antibodies.

We recently described a proteo-genomic approach for identifying sequences of antigen-specific polyclonal antibodies in animal serum (25) and reported methods for large-scale antiviral human mAb discovery from the memory B cell repertoire (26). In this study, we characterize the circulating antibody response to ebolavirus GPs at the molecular level by identifying mAbs that are present in convalescent plasma collected from an EVD survivor (**Figure 1**). For these mAbs we report the reactivity breadth, epitope specificity, prevalence in plasma, functional activities, and capacity to mediate *in vivo* protection.

## Materials and Methods 

### Human Samples

Human PBMCs and plasma were obtained at Vanderbilt University Medical Center in Nashville, TN, USA, from a survivor of the 2014 EVD epidemic after written informed consent. The studies were approved by the Vanderbilt University Medical Center Institutional Review Board. PBMCs and plasma were collected after the illness had resolved. A male human survivor of the 2014 EVD outbreak in Nigeria was age 31 when infected and age 32 when PBMCs and plasma were collected 15 months later. At time of blood collection, plasma samples were tested by qRT-PCR and found to be negative for the presence of viral RNA.

### Cell Lines

Vero-E6, Jurkat, Vero CCL-81, and THP-1 cells were obtained from the American Type Culture Collection (ATCC). Vero-E6 cells were cultured in Minimal Essential Medium (MEM) (Thermo Fisher Scientific) supplemented with 10% fetal bovine serum (FBS; HyClone) and 1% penicillin-streptomycin in 5% CO_2_, at 37°C. ExpiCHO (hamster, female origin) and FreeStyle 293F cell lines were purchased from Thermo Fisher Scientific and cultured according to the manufacturer’s protocol. The Jurkat-EBOV GP (Makona variant) cell line stably transduced to display EBOV GP on the surface (Davis et al., 2019) was a kind gift from Carl Davis (Emory University, Atlanta, GA). Jurkat-EBOV GP and THP-1 cells were cultured in RPMI 1640 (Gibco) medium supplemented with 10% FBS and 1% penicillin-streptomycin in 5% CO_2_, at 37°C.

### Viruses

The mouse-adapted EBOV Mayinga variant (EBOV-MA, GenBank: AF49101) (27), authentic EBOV Mayinga variant expressing eGFP (28), and the infectious vesicular stomatitis viruses rVSV/EBOV GP (Mayinga variant) (29) were used for mouse challenge studies or neutralization assays. Viruses were grown and titrated in Vero cell monolayer cultures.

### Monoclonal Antibodies

MAbs EBOV-515, EBOV-520, and DENV 2D22 were described previously (19). Recombinant EBOV GP-specific mAbs 13C6 and 4G7, and influenza HA-specific mAb C05 were produced as described below based on the variable gene sequences publicly available for these antibodies. Generation of new mAbs identified by proteo-genomic approach is described below.

### GP Expression and Purification

For B cell labeling, flow cytometric sorting, and purification of polyclonal antibodies from plasma, we used EBOV GP that was produced in *Drosophila* Schneider 2 (S2) cells. Briefly, recombinant ectodomain of EBOV GP ΔTM in a modified pMTpuro vector was transfected into S2 cells followed by stable selection of transfected cells with 6 μg/mL puromycin. GP ectodomain expression was induced with 0.5 mM CuSO_4_ for 4 days. Protein was purified using Strep-Tactin resin (Qiagen) *via* an engineered strep II tag and purified further by Superdex 200 (S200) column chromatography. For ELISA studies, the ectodomains of EBOV GP ΔTM (residues 1-636; strain Makona; GenBank: KM233070), BDBV GP ΔTM (residues 1-643; strain 200706291 Uganda; GenBank: NC_014373), SUDV GP ΔTM (residues 1-637; strain Gulu; GenBank: NC_006432), and MARV GP ΔTM (residues 1-648; strain Angola2005; GenBank: DQ447653) were expressed using the FreeStyle 293F cell line and purified as described before (19).

### Memory B Cell Isolation

PBMCs from a leukopak were isolated with Ficoll-Histopaque by density gradient centrifugation. The cells were cryopreserved in the vapor phase of liquid nitrogen until use. Total B cells were enriched by negative selection from PBMCs using EasySep Human Pan-B Cell Enrichment Kit (StemCell Technologies). The EBOV GP-reactive memory B cells were labeled with the recombinant EBOV GP protein that was produced in *Drosophila* S2 cells as described above and purified by flow cytometric cell sorting using an SH800 cell sorter (Sony) as described previously (6).

### Generation of Antibody Variable-Gene Libraries From Sorted B Cells

For paired antibody variable region gene sequencing, cells were resuspended into DPBS containing 0.04% non-acetylated bovine serum albumin (BSA), split into four replicates, and separately added to 50 μL of RT Reagent Mix, 5.9 μL of Poly-dt RT Primer, 2.4 μL of Additive A and 10 μL of RT Enzyme Mix B to complete the Reaction Mix, as per the vendor’s protocol. The reactions then were loaded onto a Chromium chip (10x Genomics). Chromium Single Cell V(D)J B-Cell-enriched libraries were generated, quantified, normalized and sequenced according to the User Guide for Chromium Single Cell V(D)J Reagents kits (CG000086_REV C). Amplicons were sequenced on an Illumina Novaseq 6000, and data were processed using the CellRanger software v3.1.0 (10X Genomics). Bioinformatics filtering steps were performed as described previously (26). The identities of gene segments and CDRs from germlines were determined by alignment using the ImMunoGeneTics database (30).

### Purification of GP-Reactive Polyclonal Antibodies From Plasma

Purified EBOV GP was biotinylated at a 1:20 molar ratio in PBS using EZ-Link NHS-PEG^4^-Biotin (Thermo Fisher Scientific), and then buffer-exchanged into PBS with a 0.5-mL Zeba spin column (Thermo Fisher Scientific) and coupled to Streptavidin Sepharose (GE Healthcare) at 1 mg/mL to prepare the affinity resin. Plasma was diluted 2 times in PBS, filtered through a 0.2-μm filter and applied to the 5 mL HiTrap protein G HP column (Cytiva) and the GP-reactive IgG protein fraction was purified using vendors protocol. Purified antibodies were buffer-exchanged into PBS. Approximately 1 mL of EBOV-GP-coupled resin was washed with PBS containing 0.5 M sodium chloride, loaded with PBS, and used for affinity purification of GP-reactive antibodies as follows. Unbound proteins were washed with 20 resin volumes of PBS followed by 10 resin volumes of 0.1 M glycine-HCI at pH 3.5. Bound antibodies were eluted with 5 resin volumes of 0.1 M glycine-HCI at pH 1.8 and neutralized with 1M Tris buffer to adjust the pH to 7.4. The GP-reactive IgG protein fraction was buffer-exchanged into PBS and applied to a Streptavidin Sepharose column to remove antibodies reactive to streptavidin. Antibody protein that was retained in the flow through fraction was collected, concentrated, quantified, and stored at 4°C until use.

### In-Solution Digestion and LC-MS/MS

F(ab′)_2_ fragments were prepared from purified IgG protein using IdeS cysteine protease containing polyhistidine tag (Promega) using the vendor’s protocol. F(ab′)_2_ fragments were separated from cleaved Fc fragments using protein A agarose (Pierce), and IdeS was removed using Talon Metal Affinity Resin (Takara). F(ab′)_2_ protein was buffer-exchanged into PBS and stored at -80°C until use. For mass spectrometric analysis, F(ab′)_2_ protein samples were denatured in 6 M guanidine HCl and 30 mM TCEP (tris(2-carboxyethyl)phosphine) at 60°C for 45 min. The denatured samples were alkylated with 30 mM iodoacetamide for 30 minutes in the dark. After alkylation, the samples were diluted or exchanged into appropriate buffers in accordance with the manufacture’s protocols. The manufacturer’s protocols were used for digestion conditions and enzyme ratio. Trypsin, LysC, GluC, AspN, and chymotrypsin were purchased from Promega. Elastase were purchased from Sigma Aldrich and pepsin was purchased from Worthington. All other chemicals were obtained from Sigma Aldrich. Tandem mass spectra were acquired using a Q Exactive HF Orbitrap mass spectrometer (Thermo Fisher Scientific, Bremen, Germany) modified to enable 193 nm ultraviolet photodissociation (UVPD) (31). Briefly, the instrument firmware was modified to enable triggering of an excimer laser (Excistar XS 500, Coherent, Inc.) that was aligned on-axis with the HCD cell and irradiated precursor ions trapped in the HCD cell. Peptide separations were performed using a Waters nanoAcquity UPLC (Waters Corporation). Mobile phase A and B were 0.1% formic acid in water and 0.1% formic acid in acetonitrile, respectively. Samples were prepared at 0.1 µg/µL and 5 µL was injected and washed on a trap column (10 cm x 300 µm ID, C18, packed in-house) for 5 min at 5 µL/min with 99% mobile phase A. Peptides were eluted from the trap column and separated on an analytical column (70 cm x 75 µm ID, C18, packed in-house) at 300 nL/min using a 100-minute gradient from 1% to 40% mobile phase A. Nanoflow UPLC was interfaced with the mass spectrometer using an etched fused silica electrospray emitter (360 µm OD, 20 µm ID) and electrospray voltage of 2.2 kV. Data-dependent top 5 acquisitions for HCD and UVPD used 2E5 AGC target, 30K resolving power at m/z 200, and three microscans while 1E6 AGC target, 1 microscan, and 120K resolving power at m/z 200 was used for MS1. UVPD was performed using a single laser pulse at 2 mJ, and HCD was performed with a normalized collision energy of 28. EThcD tandem mass spectra were also acquired using an Orbitrap Fusion Lumos mass spectrometer (Thermo Fisher Scientific). Data dependent EThcD acquisition parameters included MS1 resolution of 60k and AGC target of 5E5. EThcD MS2 parameters included resolution of 30k, 3 microscans, AGC target of 5E4, isolation width of 2 m/z, collision energy of 15, and a cycle time of 3 seconds.

### In-Gel Digestion and LC-MS/MS

F(ab′)_2_ protein was deglycosylated using PNGase F (New England Biolabs) per manufacturer’s protocols. 7 x 3 μg of sample was loaded into SDS-PAGE 4-12% Bis-Tris NuPage Mini-gel with the MOPS buffer system (Thermo Fisher Scientific). Light chain and heavy chain protein bands were excised for further processing using multi-enzyme digestion. Excised gel bands were washed, reduced in 10 mM dithiothreitol (DTT), alkylated in 10 mM iodoacetamide and digested. One μg of enzyme was used to digest each gel band, with the following incubation buffers: 25 mM NH_4_HCO_3_ (trypsin, chymotrypsin, and Glu-C); 50 mM Tris-HCl, pH 8.0 (elastase); 25 mM Tris-HCl, pH 8.0 (Asp-N); 25 mM Tris-HCl, 1 mM EDTA, pH 8.5 (Lys-C); 0.1% formic acid (pepsin). Arg-C was incubated in 50 mM Tris-HCl, pH 7.9, 5 mM CaCl_2_, and 2 mM EDTA followed by activation in 5 mM Tris-HCl, pH 7.9, 5 mM DTT, and 0.2 mM EDTA. All enzymes were sourced from Promega, except pepsin which was obtained from Worthington. Each gel digest was analyzed by nano LC-MS/MS with a Waters NanoAcquity HPLC system interfaced Orbitrap Velos Pro (Thermo Fisher Scientific). Peptides were loaded on a trapping column and eluted over a 75 μm analytical column at 350 nL/min; both columns were packed with Luna C18 resin (Phenomenex). The mass spectrometer was operated in data-dependent mode, with MS performed in the Orbitrap at 60,000 FWHM resolution. CID, ETD and HCD data were collected for each precursor mass, CID and ETD were collected in the ion trap and HCD data were collected in the Orbitrap at 7500 FWHM. The five most abundant ions were selected for MS/MS.

### Proteo-Genomic Data Analysis

We used the proprietary proteogenomic platform Alicanto for data analysis and visualization (25). 1,512 paired heavy and light chain antibody sequences derived from antigen-sorted memory B cells were analyzed directly by Alicanto. The tandem mass spectra dataset was mapped by Alicanto to the repertoire of paired variable heavy and light region antibody variable region sequences. Antibodies in the cDNA gene sequence repertoire were defined as being present as proteins in the plasma if there was clone-distinguishing peptide coverage over 50% of the CDR3 and full peptide coverage of the CDR3.

### Antibody Gene Synthesis

For recombinant mAb production, cDNA encoding the genes of heavy and light chains were synthesized and cloned into DNA plasmid expression vectors encoding IgG1 heavy chain and kappa or lambda light chain (32) and transformed into *E. coli* cells to produce DNA for mammalian cell expression. MAb proteins were produced following transient transfection of ExpiCHO cells following the manufacturer’s protocol and were purified as described below.

### MAb Production and Purification

For parallel production of recombinant mAbs, we used approaches designated as ‘micro-scale’ or ‘midi-scale’ (26). For ‘micro-scale’ mAbs expression, we performed transfection (~1 mL per antibody) of CHO cell cultures using a protocol for deep 96-well blocks (Thermo Fisher Scientific), as we previously described (26). For high-throughput micro-scale mAb purification, clarified culture supernatants were incubated with MabSelect SuRe resin (Cytiva), washed with PBS, eluted, buffer-exchanged into PBS using Zeba Spin Desalting Plates (Thermo Fisher Scientific) and stored at 4°C until use. For ‘midi-scale’ mAbs expression, we performed transfection (~35 mL per antibody) of CHO cell cultures as described by the vendor. MAbs were purified form culture supernatants using HiTrap MabSelect SuRe columns (Cytiva). Purified mAbs were buffer-exchanged into PBS, concentrated using Amicon Ultra-4 50 KDa Centrifugal Filter Units (Millipore Sigma) and stored at 4°C until use. To quantify purified mAbs, absorption at 280 nm (A_280_) was measured using a NanoDrop (Thermo Fisher Scientific).

### Cell-Surface-Displayed GP Antibody Binding Assays

Alexa Fluor 647 NHS ester (Thermo Fisher Scientific) was used for antibody labeling. Binding of purified polyclonal or monoclonal antibodies to Jurkat-EBOV GP or Jurkat-EBOV GP_CL_ cells was assessed by flow cytometry using an iQue Screener Plus high throughput flow cytometer (Intellicyt Corp.) as described previously (6, 19). Briefly, ˜50,000 cells were added per each well of V-bottom 96-well plate (Corning) in 5 μL of the DPBS containing 2% heat-inactivated ultra-low IgG FBS (Gibco) (designated as incubation buffer). Serial dilutions of antibody were added to the cells in replicates for a total volume of 50 μL per well, followed by 1 h incubation at ambient temperature, or 4°C in some experiments. Unbound antibody was removed by washing with 200 μL of the incubation buffer. Staining of cells was measured by flow cytometric analysis using an IntelliCyt iQue Screener Plus high throughput cytometer (Intellicyt Corp.). Data for up to 20,000 events were acquired, and data were analyzed with ForeCyt (Intellicyt Corp.) software. Dead cells were excluded from the analysis on the basis of forward and side scatter gate for viable cell population. Binding to un-transduced Jurkat cells or binding of dengue antigen-specific mAb DENV 2D22 served as negative controls for most experiments.

Cells that display cleaved GP were prepared as described previously (6, 18, 19). Briefly, Jurkat-EBOV GP cells were washed with DPBS containing calcium and magnesium (DPBS++), resuspended at 10^6^ cells/mL in DPBS containing 0.5 mg/mL of thermolysin (Promega), and incubated for 20 min at 37°C. Cleavage reaction was inhibited by washing cells with the incubation buffer containing DPBS, 2% of heat-inactivated FBS and 2 mM EDTA (pH 8.0). The GP cleavage was confirmed by loss of mAb 13C6 binding and high-level of binding that assessed with RBD-specific mAb MR78 relative to intact Jurkat-EBOV GP antibody binding. Antibody binding to un-transduced Jurkat (mock) cells served as a control for specificity of antibody staining.

For screening of the 52 micro-scale purified mAbs, cells were incubated with individual mAbs at a single 1:10 dilution, and the bound antibodies were detected using goat anti-human IgG antibody conjugated with phycoerythrin (Southern Biotech).

For the plasma antibody competition-binding assay, cells were pre-incubated with 20 µg/mL of indicated mAb for which the epitope specificity is known, followed by incubation with 20 µg/mL of Alexa Fluor 647-labeled polyclonal antibodies without washing of unlabeled mAb and flow cytometric analysis. Competition was quantified by comparing labeled polyclonal antibody binding in the presence of indicated competing mAb to the level of maximal binding estimated from binding of labeled polyclonal antibodies in the presence of the dengue virus-specific mAb DENV 2D22.

For the epitope mapping competition-binding assay, cells were pre-incubated with 20 µg/mL of purified mAb followed by incubation with 2 µg/mL of Alexa Fluor 647-labeled mAb for which the epitope specificity is known and flow cytometric analysis. Competition was quantified by comparing labeled mAb binding in the presence of indicated competing mAb to the level of maximal binding estimated from binding of labeled mAb alone. Tested mAbs were considered competing if their presence reduced the reference antibody binding to less than 30% of its maximal binding.

### ELISA Binding Assays

Wells of 96-well microtiter plates were coated with 1 µg/mL of purified recombinant GP (produced in the FreeStyle 293F cell line) in DPBS at 4°C overnight. Plates were blocked with 2% non-fat dry milk (Bio-Rad Laboratories) and 2% normal goat serum (Gibco) in DPBS containing 0.05% Tween-20 (DPBS-T) for 1 h. For rapid screening analysis, micro-scale purified mAbs were assessed at single 1:10 dilution in blocking buffer, added to the wells and incubated for 1 h at ambient temperature. The bound antibodies were detected using goat anti-human IgG conjugated with horseradish peroxidase (Southern Biotech) and TMB substrate (Thermo Fisher Scientific). Color development was monitored, 1N hydrochloric acid was added to stop the reaction and the absorbance was measured at 450 nm using a spectrophotometer (Biotek).

For dose-response assays, serial dilutions of plasma of purified polyclonal antibodies were applied to antigen-coated wells in triplicate, and the assay was performed as described above. Non-linear regression analysis was used for curves fitting.

### Epitope Mapping Using an EBOV GP Alanine-Scan Mutation Library

Epitope mapping was carried out essentially as described previously (33) using an alanine-scan mutation library of EBOV GP (Yambuku-Mayinga variant; Uniprot accession number Q05320) lacking the mucin-like domain (residues 311-461). Our previous mapping of almost 200 anti-EBOV mAbs identified 131 mutant GP clones validated as representing critical epitope residues. These GP cDNA clones were arrayed into 384-well plates, one mutant per well, transfected into HEK-293T cells and allowed to express for 22 hours. Cells were fixed in 4% paraformaldehyde in PBS containing calcium and magnesium and incubated with antibody diluted in 10% normal goat serum (Sigma-Aldrich) for 1 h at ambient temperature, followed by a 0.5 h incubation with Alexa Fluor 488-conjugated secondary antibodies (Jackson ImmunoResearch Laboratories) in 10% normal goat serum. Cells were washed twice with PBS without calcium or magnesium and resuspended in CellStripper (Cellgro) containing 0.1% BSA (Sigma-Aldrich). Cellular fluorescence was detected using an Intellicyt high throughput flow cytometer. Background fluorescence was determined by fluorescence measurement of vector-transfected control cells. MAb reactivities against each mutant EBOV GP clone were calculated relative to wild-type EBOV GP reactivity by subtracting the signal from mock-transfected controls and normalizing to the signal from wild-type GP-transfected controls. Mutated residues within critical clones were identified as critical to the mAb epitope if they did not support reactivity of the test mAb but did support reactivity of other control EBOV mAbs.

### Neutralization Assays

A virus neutralization screening assay was performed under maximum biosafety level 4 (BSL-4) containment using recombinant EBOV-eGFP virus, as described previously (34). Briefly, mAbs were mixed with virus and applied to Vero-E6 cell monolayer cultures. In the absence of mAb neutralizing activity, the infection resulted in uniform eGFP fluorescence from the monolayer of cells that was detected readily by fluorescence microscopy. For rapid screening analysis, micro-scale-purified mAbs were tested at a single 1:6 dilution; concentrations were not normalized. The results were expressed as percent virus neutralization relative to the infected cells control.

Dose-response mAb neutralization studies were performed using a plaque reduction neutralization test using infectious recombinant rVSV/EBOV-GP or rVSV/EBOV-GP_CL_, as described previously (19). rVSV/EBOV-GP_CL_ was generated by rVSV/EBOV-GP treatment with thermolysin (Promega), as described previously (19). MAbs were tested at four-fold dilutions starting at 200 µg/mL in triplicate. Half maximal inhibitory concentration (IC_50_) values were determined by nonlinear regression analysis using Prism software.

### Antibody-Mediated Cellular Phagocytosis by Human Monocytes

The assay was performed as described before using EBOV GP-coupled Alexa Fluor 488 Neutravidin beads and the THP-1 cell line (19). Micro-scale-purified mAbs were tested at a single 1:6 dilution; concentrations were not normalized. Results were expressed as a phagocytic score that was determined using the percentage of Alexa Fluor 488^+^ cells and the median fluorescence intensity (MFI) of the Alexa Fluor 488^+^ cells. A recombinant antibody based on the variable gene sequences of EBOV GP-specific mAb 13C6 was used as a positive control, and a recombinant antibody based on the variable gene sequences of the influenza virus A hemagglutinin-specific mAb C05 was used as a negative control.

### Mouse Challenge

Seven- to eight-week old female BALB/c mice were obtained from the Jackson Laboratory. Mice were housed in microisolator cages and provided food and water *ad libitum*. Challenge studies were conducted under maximum containment in an animal biosafety level 4 (ABSL-4) facility of the Galveston National Laboratory, UTMB. Groups of mice (n = 5 per group) were inoculated with 1,000 PFU of the EBOV-MA by the intraperitoneal (i.p.) route. Mice were treated i.p. with 75 μg per mouse of individual mAbs on the first day after virus inoculation (dpi). Antibody DENV 2D22 served as a control. Mice were monitored twice daily from 0 to 14 dpi for illness, survival, and weight loss, followed by once daily monitoring from 15 dpi to the end of the study at 28 dpi. The extent of disease was scored using the following parameters: score 1 – healthy; score 2 – ruffled fur and hunched posture; score 3 – a score of 2 plus one additional clinical sign such as orbital tightening and/or >15% weight loss; score 4 – a score of 3 plus one additional clinical sign such as reluctance to move when stimulated, or any neurologic signs (seizures, tremors, head tilt, paralysis, etc.), or >20% weight loss. Animals reaching a score of 4 were euthanized as per the IACUC-approved protocol. All mice were euthanized on day 28 after EBOV challenge.

### Statistical Analysis

The descriptive statistics mean ± SEM or mean ± SD were determined for continuous variables as noted. Survival curves were estimated using the Kaplan-Meier method and curves compared using the two-sided log rank test (Mantel-Cox) with subjects right censored, if they survived until the end of the study. To correct for multiple comparisons Bonferroni-corrected threshold for significance level was determined. The comparisons for plasma antibody competition-binding assay was performed using ordinary one-way ANOVA with Dunnett’s multiple comparisons test. In neutralization assays, IC_50_ values were calculated after log transformation of antibody concentrations using a four-parameter log-logistic (4PL) analysis. Technical and biological replicates are indicated in the figure legends. Statistical analyses were performed using Prism v8.4.3 (GraphPad).

## Results

### Proteo-Genomic Identification of Ebolavirus Glycoprotein-Specific mAbs From Human Plasma

**Figure 2:**
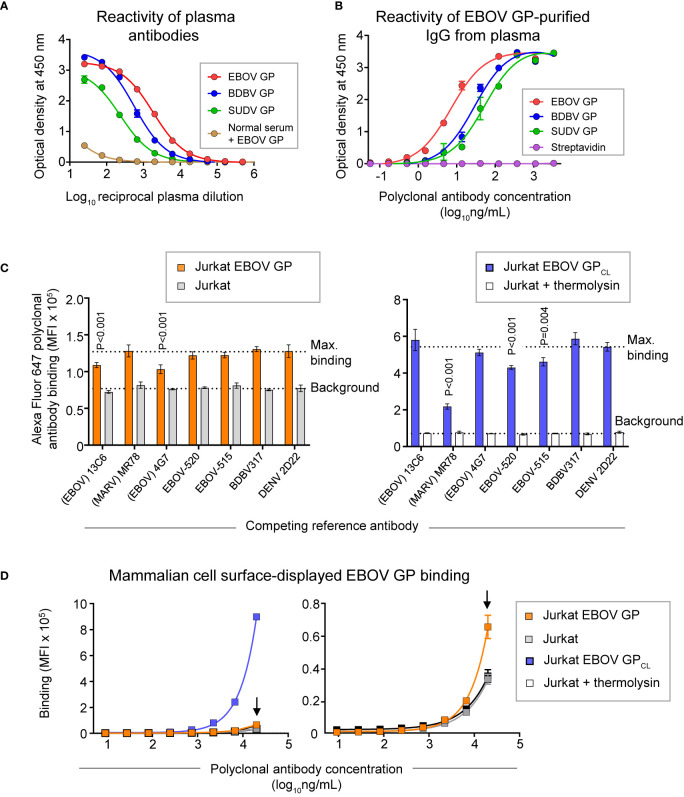
GP recognition, reactivity breadth, and competition binding profiles identified for polyclonal antibody response in convalescent plasma from an EVD survivor. **(A)** Dose-response binding of antibodies in the survivor’s plasma to recombinant EBOV, BDBV, or SUDV GP proteins was assessed by ELISA. Mean ± SD of technical triplicates from one experiment are shown. Binding of serum antibodies to EBOV GP from an individual without prior EBOV exposure served as the control for the level of specific binding. **(B)** Dose-response binding of polyclonal IgG antibodies that were affinity purified from plasma tested in panel **(A)** using an EBOV GP-coupled column was assessed by ELISA using recombinant EBOV, BDBV, or SUDV GP proteins. Mean ± SD of technical triplicates from one experiment are shown. **(C)** Competition-binding analysis of Alexa Fluor 647-labeled purified polyclonal antibodies to mammalian cell surface-displayed GP. Antibodies were purified from plasma as in panel **(B)** and labeled with Alexa Fluor 647. Jurkat-EBOV GP cells (left) or Jurkat-EBOV GP cells that were pre-treated with thermolysin to generate cleaved GP (designated as Jurkat-EBOV GP_CL_ [right]) were pre-incubated with in the presence of indicated monoclonal antibody with known epitope specificity followed by incubation with fluorescently-labeled polyclonal antibodies and flow cytometric analysis. Competition was quantified by comparing labeled polyclonal antibody binding in the presence of indicated competing mAb to the level of maximal binding (upper dotted line) estimated from binding of labeled polyclonal antibodies in the presence of the dengue virus-specific antibody DENV 2D22. Binding to untransduced (mock) Jurkat cells (open bars) served as control for the background (lower dotted line). Mean ± SD of technical duplicates from two experiments are shown. Binding in the presence of each indicated mAb was compared to the maximal binding control using ordinary one-way ANOVA with Dunnett’s multiple comparisons test, and P-values for significant differences are indicated. **(D)** Dose-response binding curves of purified fluorescently-labeled polyclonal IgG to Jurkat-EBOV or Jurkat-EBOV GP_CL_ cells determined by flow cytometric analysis. Mean ± SD of technical triplicates from one experiment are shown.

Peripheral blood mononuclear cells (PBMCs) and plasma were collected from a human survivor of the 2014 EVD outbreak in Nigeria 15 months after infection. Plasma from this individual exhibited a high level of GP-specific binding and broad reactivity for diverse ebolavirus species as measured by ELISA using EBOV, BDBV, and SUDV recombinant soluble GP proteins (containing the full extracellular domain but lacking a transmembrane domain) (**Figure 2A**). In two separate studies, we identified sequences of ebolavirus GP-specific antibodies from circulating plasma IgG protein and memory B cells.

Total plasma IgG protein was purified using a protein G-coupled affinity chromatography column followed by ebolavirus-reactive IgG purification using an EBOV GP-coupled affinity column. EBOV GP-purified polyclonal IgG demonstrated high specificity and broad reactivity to the three ebolavirus GPs by ELISA (**Figure 2B**) similarly to the GP reactivity and breadth identified in the whole plasma IgG binding assay (**Figure 2A**). We next defined groups of GP-specific antibodies that bound to common major antigenic sites in the purified EBOV GP-reactive IgG fraction. We used a competition-binding assay with Jurkat cells stably transduced to express EBOV GP on their surface (Jurkat-EBOV GP) or the same cells that had been treated with thermolysin to generate cell surface-displayed proteolytically cleaved GP (Jurkat-EBOV GP_CL_). Cells were pre-incubated with EBOV-GP-reactive mAbs for which the epitope specificity is known, including antibodies that recognize glycan cap (13C6) (33), base region (4G7, EBOV-515, and EBOV-520) (19, 33), receptor binding site (MR78 that is specific to Marburg virus [MARV] GP and recognize EBOV GP_CL_) (35, 36), stalk (BDBV317), or mAb DENV 2D22 specific to dengue virus (37). The level of competition binding was estimated using fluorescently-labeled EBOV-GP-purified plasma IgG by comparing to binding in presence of DENV 2D22. This study revealed that polyclonal antibody responses in this survivor targeted glycan cap and base region epitopes on intact GP and the GP base and RBS regions on GP_CL_, with a high prevalence of base- and RBS-specific antibodies directed against GP_CL_ (**Figure 2C**). Binding of EBOV-GP-purified polyclonal IgG to Jurkat-EBOV GP or GP_CL_ demonstrated that most reactivity in the plasma is mediated by antibodies recognizing epitopes on GP_CL_ (**Figure 2D**).

F(ab′)_2_ fragments were prepared from the purified EBOV GP-reactive IgG protein fraction using IdeS cysteine protease that digests antibodies at a specific site below the hinge. The resulting fragments were subjected to high-resolution liquid chromatography coupled to tandem mass-spectrometry. Multiple in-gel and in-solution protease digestion products were analyzed with higher energy collisional dissociation/ultraviolet photodissociation (HCD/UVPD) on a customized Thermo Q Exactive HF and EThcD on a Orbitrap Fusion Lumos mass spectrometer (Thermo Fisher Scientific). Bottom-up and middle-down MS/MS spectra yielded peptides between 6 and 40 amino acid of length. From 60 mL of plasma, we obtained ˜0.16 mg of EBOV-GP-reactive F(ab′)_2_ fragments that resulted in >205,260 mass spectra and 42 h of LC-MS/MS time.

In parallel study, circulating B cells were enriched from PBMCs by negative selection using magnetic beads (STEMCELL Technologies). EBOV GP-reactive CD19^+^ B cells were identified after labeling with biotinylated recombinant soluble EBOV GP protein followed by detection with allophycocyanin-conjugated streptavidin. EBOV-GP-labeled B cells were isolated by sorting in bulk using a Sony flow cytometer. Isolated antigen-specific B cells were loaded on a microfluidics device for single cell partitioning and barcoding (Chromium Controller; 10X Genomics) followed by reverse transcription with PCR and sequence analysis to obtain paired heavy and light chain antibody variable gene sequences. A detailed protocol for this antibody discovery workflow has been described previously (26). From ˜10^8^ PBMCs we sorted ∼20,000 EBOV GP-reactive B cells and identified 1,512 paired antibody heavy and light chain variable region sequences (**Table S1**).

Using the Alicanto proteo-genomic analysis approach (25), we next determined the sequences of protein antibodies in plasma that were shared between plasma IgG and memory B cell antibody variable gene repertoires. Antibodies in the repertoire were identified as being present in the plasma if there was clone-distinguishing peptide coverage over 50% of the complementarity determining region 3 (CDR3) and general peptide coverage over 100% of the CDR3 (**Figure S1**). We compared the amino acid sequences from the plasma proteomics experiments with the inferred amino acid sequences based on paired cDNA antibody sequences from memory B cells in the database obtained with the 10X Genomics single-cell experiments. This approach identified 5 individual heavy and 48 individual light chain variable region sequences in plasma (for a total of 53 heavy or light chain sequences), that were ranked based on the distinct peptide count for peptides covering the corresponding CDR3 (**Table S2**). Heavy chain variable region CDR3s had between 1 to 15 CDR3-covering peptides, and light chain CDR3s had between 1 to 10 peptides (**Table S2**). Of the 53 heavy or light chain variable region protein sequences identified in plasma, we identified one cognate pair for which both the heavy and light chain proteins were both detected in plasma and also found as paired cDNA sequences from a single circulating memory B cell. For the remaining 4 heavy or 47 light chain variable region protein sequences that were identified in plasma, we identified either a matching heavy or light chain cDNA sequence in the single-cell paired sequence database. Therefore, we identified a panel of 52 individual mAbs that we identified in both plasma and memory B cells with at least one chain match (**Table S3**). While the overall complexity of plasma and memory B cell repertoires was difficult to assess, the overlap between these two repertoires was small as evidenced by the small number of proteomically-identified sequences and the small number of heavy chain variable region CDR3s compared to the number of light chain variable region CDR3s identified. Thus, only a small portion of the large memory B cell antibody repertoire we obtained was detected as IgG protein in the plasma from this survivor at the depth of proteomics and genomics analysis we achieved.

### MAbs Identified in the Proteo-Genomic Approach Recognize Ebolavirus GP, Demonstrate Diverse Reactivity to the GPs of Three Medically Important Ebolaviruses, and Exhibit Varying Fv- and Fc Region-Mediated Functional Activities

**Figure 3:**
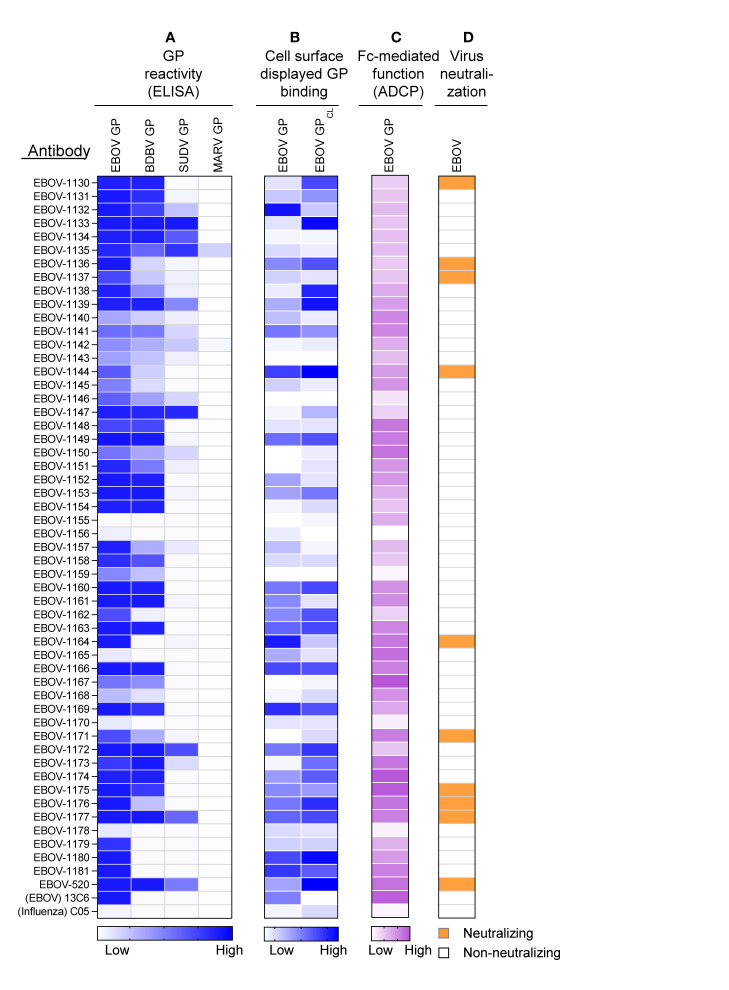
Reactivity and functional activity of 52 monoclonal antibodies identified in plasma by proteomic studies. Antibody sequences that were present in both genomic and proteomic repertoires were determined, generated as synthetic antibody-encoding cDNA in mammalian immunoglobulin IgG expression vectors, and produced as recombinant IgG1 using transiently-transfected CHO cells and a micro-scale expression protocol. Micro-scale purified antibodies were assessed for binding and functional activities at a single dilution (see Methods and **Table S4**). The broadly reactive neutralizing mAb EBOV-520, and recombinant forms of EBOV GP monospecific mAb 13C6 and influenza A hemagglutinin-specific mAb C05 were used as controls. **(A)** MAb binding to each of four GP proteins, including EBOV, BDBV, SUDV, and MARV GP. The figure shows a heatmap for binding of 52 mAbs expressed recombinantly, representing optical density (O.D.) values measured at 450 nm for each antigen (range, 0.1 – 3.7). White indicates a lack of detectable binding, while blue indicates moderate binding and darker blue indicates higher binding. **(B)** MAb binding to Jurkat-EBOV or Jurkat-EBOV GP_CL_ cells determined by flow cytometric analysis. The figure shows a heatmap for binding of 52 tested mAbs, representing log_10_ median fluorescence values for each tested condition (range, 3.6 – 6.4). White indicates background binding, and the gradient of blue indicates moderate binding and darker blue indicates the extent of specific binding. Binding of human antibody was detected using secondary goat anti-human IgG-specific antibodies conjugated to phycoerythrin. **(C)** Antibody Fc region-mediated cellular phagocytosis activity determined using EBOV-GP-coupled fluorescent beads and THP-1 monocyte human cell line. The figure shows a heatmap for binding of 52 tested mAbs, representing phagocytic score values (range, 62 to 195). **(D)** Screening for neutralizing antibody activity using biosafety level 4 recombinant EBOV encoding enhanced green fluorescent protein (eGFP). Green indicates neutralizing activity (>30% virus neutralization at the single mAb dilution tested), and white designates lack of neutralizing activity (<30% virus neutralization at the single mAb dilution tested).

The mAbs identified in the approach described above were produced as recombinant IgG1 in transiently-transfected Chinese hamster ovary (CHO) cells for functional antibody analysis using previously described high-throughput approaches for rapid antibody production and purification from small sample volumes designated as microscale (26). Fifty-one of 52 recombinant antibodies expressed sufficiently well to characterize their activity (**Table S4**). For initial screening purposes, each mAb was tested at a single dilution from microscale purification. The mAb concentration ranged from 1 to 60 μg/mL for GP binding assays, and from 2 to 100 μg/mL for *in vitro* functional assays. The reactivity of individual mAbs was assessed by ELISA using recombinant EBOV, BDBV, or SUDV GP proteins. All identified mAbs exhibited ebolavirus GP-specific binding and revealed diverse reactivity profiles, in which the majority of mAbs reacted to EBOV or EBOV and BDBV GP, and a smaller fraction of mAbs cross-reacted to the GP of all three ebolaviruses (**Figure 3A**). Binding analysis using cell-surface-displayed EBOV GP of EBOV GP_CL_ suggested recognition of diverse epitopes on the GP (**Figure 3B**). We next assessed the functional activities of mAbs by performing Fc-mediated effector function and virus neutralization *in vitro* assays, because the activities measured by these assays may contribute to *in vivo* antibody function. We performed an antibody-dependent cellular phagocytosis (ADCP) assay that used beads coupled with recombinant EBOV GP to determine the capacity of bound mAb to activate human effector cells *in vitro*. ADCP profiling revealed diverse activation patterns with low, intermediate, or high activities observed for individual mAbs (**Figure 3C**). A relatively small fraction (9 of 52) of mAbs from the panel possessed detectable neutralizing activity against live EBOV (**Figure 3D**) based on a >30% virus neutralization cutoff for a single tested mAb dilution (**Table S4**). The reactivity of most (8 of 9) neutralizing mAbs we identified was limited to the homologous EBOV GP or EBOV and BDBV GP, and one neutralizing mAb cross-reacted to GPs of all three tested ebolaviruses. Several non-neutralizing mAbs bound efficiently to cell-surface-displayed GP and exhibited high ADCP activity, suggesting these mAbs may also contribute to protective immunity. Together, these studies revealed diverse patterns of recognition of ebolavirus GP by mAbs that were present in the plasma of the EVD survivor and identified functional mAbs that possessed neutralizing and/or the Fc-mediated effector function activities.

### Major Antigenic Binding Sites Targeted by Class-Representative Plasma mAbs Revealed Common Features

**Figure 4:**
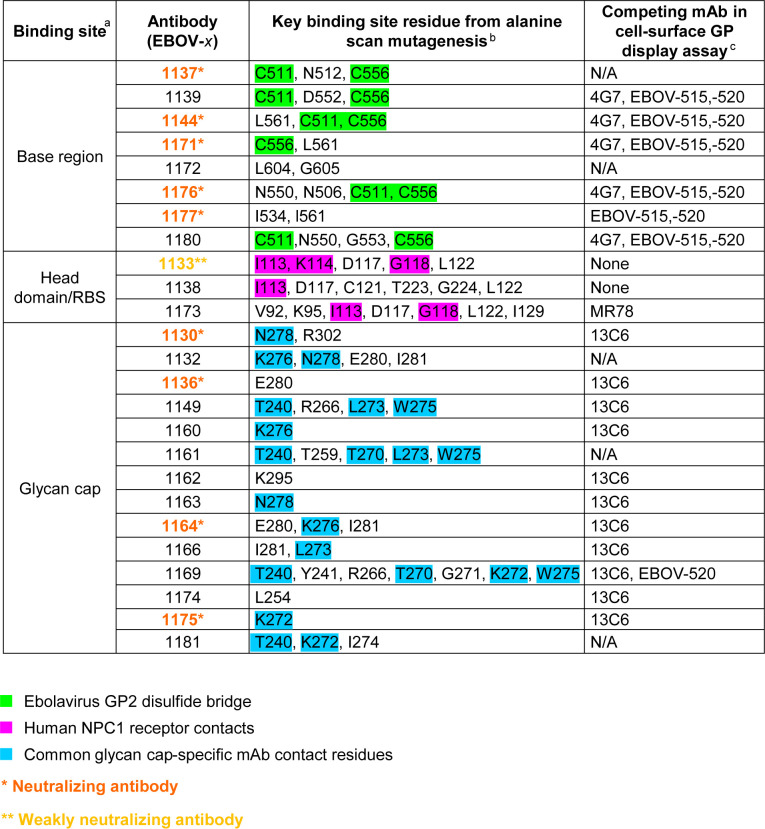
Epitope mapping identified common features of plasma antibodies recognizing three major antigenic sites on EBOV GP. ^a^Major antigenic sites that were recognized by plasma-represented mAbs include base region, head domain/receptor binding site (RBS), and glycan cap. ^b^GP mutations that reduce binding for indicated antibodies to the GP identified by alanine-scanning mutagenesis of cell surface-displayed EBOV GP library. Amino acids and their positions for key binding site residues are indicated. Residues that form a disulfide bridge in the GP subunit near the internal fusion loop (IFL)-heptad repeat 1 (HR1) interface (green), the Niemann-Pick C1 (NPC1) receptor binding site residues (magenta), and residues of the glycan cap that are commonly recognized by glycan cap-specific mAbs are (blue) are highlighted in respective colors. ^c^Competition-binding was assessed by measuring binding of fluorescently-labeled antibodies to Jurkat-EBOV GP cells that were pre-incubated with indicated unlabeled competing antibody. Competition was defined as <30% of labeled antibody binding in the presence of respective competing mAb relative to a non-competing mAb control. N/A indicates Not Available, for cases in which the antibody exhibited poor solubility after production or after fluorescent labeling and has not been tested in competition-binding studies. "None" indicates antibody did not compete with any of tested reference antibodies.

To determine the molecular features of GP recognition by representative plasma antibodies, we produced 25 mAbs from the panel in a larger scale. For this study, we selected all of the neutralizing mAbs and also mAbs that demonstrated strong binding to cell-surface-displayed EBOV GP or EBOV GP_CL_ (**Figure 3**). Previous findings by us and others showed that efficient binding to cell-surface-displayed GP is often associated with antibody protective functions (18, 19). To define key contact residues in the binding site, we used alanine scanning mutagenesis of the GP and tested the binding of individual mAbs to each member of a shotgun mutagenesis alanine mutation library of the EBOV GP displayed in cells. In addition, we performed competition-binding analysis using reference EBOV-GP-reactive mAbs for which the epitope specificity is known and cell-surface-displayed EBOV GP to identify major binding sites recognized by the tested mAbs (**Figure 4**). This analysis revealed that the representative plasma mAbs recognized three major binding sites, which included the base region (8 mAbs), the head domain/RBS region (3 mAbs), and the glycan cap (14 mAbs).

**Figure 5:**
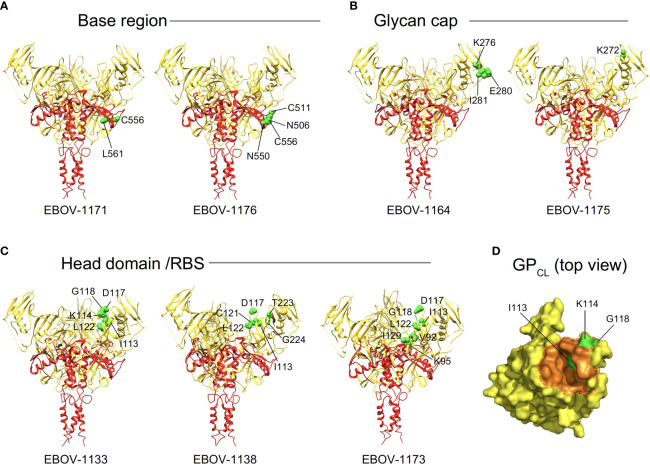
Epitope residues of class representative plasma antibodies recognizing three major antigenic sites on EBOV GP. Amino acids and their positions for key binding site residues (green) are indicated for representative base-specific **(A)**, glycan cap-specific **(B)**, or head domain/RBS-specific **(C)** antibodies and shown on a ribbon diagram of the EBOV GP trimer (PDB ID: 5JQ3) for one protomer. GP1 is in yellow, and GP2 is in red. **(D)** The NPC1 receptor binding site residues of the RBS (PDB ID: 5F1B) are shown in orange and NPC1 contact region is indicated within a dashed orange shape on GP_CL_ (top view). RBS residues I113, K114, and G118 identified as critical for binding of head domain/RBS-specific antibodies are shown in green.

Of the 9 strongly neutralizing mAbs in this panel, 5 mapped to the base region of the GP (**Figure 4**), along with 3 non-neutralizing mAbs. Most of the base-specific mAbs (7 of 8) exhibited stronger binding to EBOV GP_CL_ than to uncleaved EBOV GP (**Figure 3B**), which was reminiscent of the binding pattern of polyclonal antibodies to cleaved GP (**Figures 2C, D**), although these mAbs also bound strongly to intact cell-surface-displayed GP. The majority (4 of 5) of neutralizing base-region-specific mAbs bound strongly to EBOV but not to BDBV or SUDV GP. One neutralizing mAb (EBOV-1177) cross-reacted strongly to the GP of all three ebolaviruses (**Figure 3A**), suggesting that this mAb could mediate broad neutralizing activity. Another common feature of these class-representative mAbs was the similar location of key binding site residues. These included residues positioned near the IFL-heptad repeat 1 (HR1) interface, and GP2 residues N550, D552, G553, C511 and C556 (which form a disulfide bridge) that were identified previously as key binding site residues for the potently neutralizing base-region murine mAbs 2G4 and 4G7 (**Figures 4**, **5A**) (33). MAbs 2G4 and 4G7, with the glycan cap-specific mAb 13C6, formed the basis of therapeutic three-antibody cocktail ZMapp™ used for treatment of human EVD (8, 9). In agreement with the epitope residues mapped using alanine scan mutagenesis studies, the base-specific mAbs competed with mAb 4G7 and/or the base-specific mAbs EBOV-515 or EBOV-520 for the GP binding, as was shown by the cell-surface-display GP assay (**Figure 4**).

Another class of EBOV GP-reactive IgGs that was highly-represented in plasma comprised of mAbs that recognize the glycan cap (**Figure 4**). Several previous studies postulated an indispensable role in EBOV protective mAbs for Fc-mediated effector functions of glycan-cap-specific mAbs that may be sufficient alone or complement neutralizing activity (21, 38, 39). Of 14 characterized glycan cap-specific mAbs, most demonstrated strong reactivity to both EBOV and BDBV GP by ELISA, many mAbs exhibited moderate or high Fc-mediated effector function activities, and four mAbs neutralized live EBOV (**Figures 3A, C, D**), suggesting these mAbs would have *in vivo* functions. For individual mAbs, alanine scanning mutagenesis studies identified key binding site residues that mostly were located on the top part of the glycan cap (**Figures 4**, **5B**). Many of these residues were defined previously by us and others as key contact residues for potent neutralizing glycan cap-specific human mAbs BDBV-289 (W275), EBOV-237 (N278), EBOV-337 (W275), EBOV-442 (W275, P273), and EBOV-548 (T240, T270, I274, W275), weakly neutralizing protective murine mAb 1H3 (K276), or the non-neutralizing protective mAb 13C6 (T270, K272) (6, 33, 40, 41). We also assessed mAbs from this study in a competition-binding assay and demonstrated competition with mAb 13C6 for GP binding, which confirmed the glycan cap specificity of these mAbs (**Figure 4**).

Ebolavirus entry involves cathepsin-mediated cleavage of GP into GP_CL_ in the endosome (42). Cleavage removes the glycan cap and mucin-like domain of GP ectodomain, thereby exposing the RBS for engagement of the NPC1 in the endosome (43, 44). The other highly-represented class of EBOV GP-reactive IgGs in plasma included mAbs that recognize head domain/RBS. These Abs exhibited broader reactivity to recombinant diverse GPs when compared to those identified for the base-region specific class of mAbs (**Figures 3A**, **4**). Of three characterized mAbs, two were non-neutralizing and one mAb (EBOV-1133) possessed weak neutralizing activity (**Figure 4**). Lack of potent virus neutralization by this class of mAbs likely is explained by recognition of cryptic epitopes on the intact cell-surface-displayed GP. Thus, all three mAbs bound preferentially to cleaved GP (Jurkat-EBOV GP_CL_) and showed weak or no detectable binding to uncleaved cell-surface-displayed GP (**Figure 3B**), in a binding pattern similar to that of polyclonal antibodies to cleaved GP (**Figures 2C, D**). Alanine scanning mutagenesis studies identified that, in addition to the binding site residues for these mAbs located on the GP head, three residues (I113, K114, and G118) mapped to a place in the RBS that is exposed only after GP cleavage (**Figures 4**, **5C, D**). In agreement with these results, one identified mAb (EBOV-1173) competed for binding to GP_CL_ with the RBS-specific Marburg virus (MARV) GP mAb MR78 that recognizes not only MARV GP but also EBOV GP_CL_ (36). Two other mAbs likely have distinct epitopes within the RBS, such as they did not compete with MR78 (**Figure 4**).

Together, these studies revealed that antibodies recognizing three major antigenic sites - base region, glycan cap, and head domain/RBS - dominate the convalescent plasma IgG protein repertoire of the EVD survivor and suggested preferential “hot spot” binding site residues on the GP for recognition by class-representative plasma mAbs.

### Class-Representative Plasma mAbs Exhibited Varying Levels of Neutralizing Activity Against Chimeric VSV Displaying GP or GP_CL_

**Figure 6:**
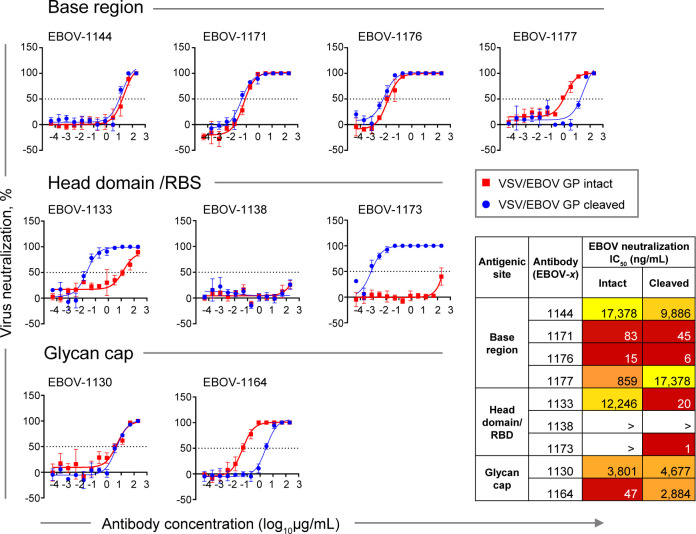
Neutralizing potency by class-representative plasma antibodies. Dose-response neutralization of recombinant infectious vesicular stomatitis virus (VSV) expressing EBOV GP in place of the endogenous VSV glycoprotein (VSV/EBOV GP). Curves for intact VSV/EBOV GP neutralization are shown in red, and curves for thermolysin-cleaved VSV/EBOV GP neutralization that generates virus surface-exposed cleaved GP (defined as VSV/EBOV GP_CL_) are shown in blue. Half maximal inhibitory concentration (IC_50_) _was_ estimated using nonlinear regression analysis. Mean ± SD of technical triplicates from one experiment are shown.

Given many mAbs differentially recognized GP and GP_CL_ in our Jurkat cell surface-displayed GP binding screening assay, we next examined in more detail the neutralizing capacity of class-representative mAbs (**Figure 6**). We used a replication-competent recombinant vesicular stomatitis virus (rVSV) displaying a full-length EBOV GP in a place of VSV glycoprotein (rVSV/EBOV-GP) or thermolysin-treated rVSV/EBOV-GP that generates a cleaved intermediate form of GP (GP_CL_) displayed on the virion surface (rVSV/EBOV-GP_CL_) to estimate dose-response neutralization by class-representative mAbs. We assessed four base region-specific mAbs and two glycan cap-specific mAbs that were selected based on their capacity to neutralize live EBOV in the initial screening study and all three head domain/RBS-specific mAbs that did not show detectable EBOV neutralization (**Figure 3**).

Base region-specific mAbs, in agreement with their ability to efficiently recognize both Jurkat cell surface-displayed GP and GP_CL_, showed similar neutralization activity for both rVSV/EBOV-GP and rVSV/EBOV-GP_CL_ (**Figure 6**, top panel), indicating that antigenic sites for this class of mAbs are accessible on intact GP for antibody binding and neutralization. Antibodies EBOV-1171 and EBOV-1176 potently neutralized rVSV/EBOV-GP with half maximal inhibitory concentration (IC_50_) values below 100 ng/mL, indicating the presence of high-potency neutralizing mAbs in the plasma of this EVD survivor.

In contrast, two of three head domain/RBS that recognized cell surface-displayed GP_CL_ only and did not neutralize EBOV, exhibited a large (˜600-fold or higher) increase in neutralizing potency against VSV displaying cleaved GP, and partial (40 to 90%) neutralization of rVSV/EBOV-GP at the highest concentration tested (200 μg/mL) (**Figure 6**, middle panel**)**. We concluded that the antigenic sites for these two mAbs are occluded on intact GP and become accessible only after proteolytic priming in the endosome of infected cells.

Two EBOV neutralizing mAbs that recognize the glycan cap, EBOV-1130 or EBOV-1164, neutralized rVSV/EBOV-GP with high (IC_50_ = 47 ng/mL) or moderate (IC_50_ = 3,801 ng/mL) potency, respectively (**Figure 6**, bottom panel). EBOV-1164 neutralized rVSV/EBOV-GP_CL_ ∼60-fold less efficiently when comparing the IC_50_ value to the corresponding IC_50_ value from neutralization of rVSV/EBOV-GP by this mAb, indicating that the binding site for EBOV-1164 is largely altered after GP cleavage. Neutralization of rVSV/EBOV-GP and rVSV/EBOV-GP_CL_ by EBOV-1130 was similar, indicating that thermolysin treatment did not alter the binding site for this mAb.

Given that only extracellular virus is accessible for antibody recognition (and contains full-length intact GP), these studies suggest that neutralizing activity in the plasma of this EVD survivor is mediated principally by potent base region-specific mAbs and, to a lesser extent, glycan cap-specific mAbs.

### Class-Representative Plasma mAbs Showed Varying Levels of *In Vivo* Activity to Protect Mice Against Lethal EBOV Challenge

**Figure 7:**
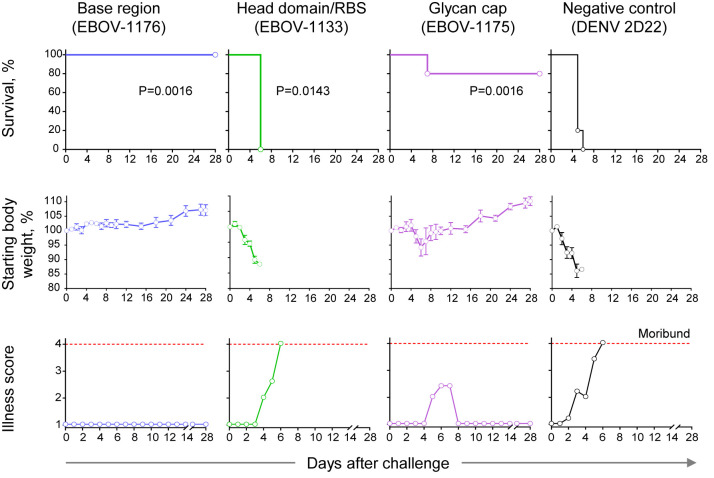
Therapeutic protection by class-representative plasma antibodies in mice. *In vivo* therapeutic protection by individual antibodies. C57BL/6 mice (n = 5 per group) were challenged with mouse-adapted EBOV-MA, treated at 1 day after virus inoculation with indicated antibody at a dose of 3.75 mg/kg, and monitored for 28 days. Dengue virus human antibody DENV 2D22 served as a control. Survival curves (top panel) were estimated using the Kaplan-Meier method, and each treatment group was compared to the DENV 2D22 group using a two-sided log-rank (Mantel–Cox) test. P-values are indicated for each comparison, and Bonferroni-corrected threshold for significance level was set up to P = 0.017. Weight change (middle panel) and illness score (bottom panel) are shown. Data represent one experiment.

We next assessed the protective capacity of class-representative mAbs (choosing one for each of the groups recognizing the three major binding sites) using a stringent *in vivo* mouse virus challenge model (**Figure 7**). These mAbs included the base region-specific mAb EBOV-1176 that possessed high neutralizing activity (**Figures 3D**, **6**), the head domain/RBS-specific mAb EBOV-1133 that weakly neutralized rVSV/EBOV-GP and strongly neutralized rVSV/EBOV-GP_CL_ (**Figure 6**), and the glycan cap-specific mAb EBOV-1175 that showed high ADCP activity in addition to capacity to neutralize live EBOV (**Figures 3C, D**). An irrelevant antibody DENV 2D22 (IgG1 isotype) specific to dengue virus served as a control. We challenged groups of mice with mouse-adapted EBOV (EBOV-MA) on day 0 and administered mAbs at a dose of 75 μg per mouse (∼3.75 mg/kg) one day later (1 dpi). DENV 2D22-treated animals succumbed to the disease by 6 dpi (median survival, 5 dpi). Base region-specific mAb EBOV-1176 conferred full protection from morbidity, weight loss, and illness, demonstrating the high potency of this class-representative antibody. High *in vivo* potency had been reported previously for many base region-specific mAbs isolated from memory B cells (18, 19, 21, 23, 45–47), indicating that this region is a major site of vulnerability on ebolavirus GP for neutralizing antibodies. Treatment with head domain/RBS-specific mAb EBOV-1133 delayed mortality (median survival, 6 dpi; P=0.0143, by the Mantel-Cox test) when compared with the control group of mice but did not protect animals. The glycan cap-specific mAb EBOV-1175 treatment partially protected animals from death (80% survival; P=0.0143, by the Mantel-Cox test), severe weight loss, and illness when compared to the control group of mice. Intermediate-to-low *in vivo* potency, which is likely mediated by a combination of neutralizing and Fc-mediated effector function antibody activities, had been reported previously for glycan cap region-specific mAbs isolated from memory B cells (21, 22, 39). Thus, these studies define the glycan cap as the other major site of vulnerability on ebolavirus GP. Together these results suggested that the protective activity in the plasma of this EVD survivor is likely mediated by highly-represented and potent base region-specific antibodies and, at lesser extent, by antibodies recognizing the glycan cap of the GP.

## Discussion

As new pathogens and vaccines to combat these pathogens emerge, a comprehensive molecular analysis of protective polyclonal antibody responses in serum becomes critical for defining determinants of antibody-mediated immunity. A methodology for determining the antibody composition of the polyclonal serum response was described in pioneering studies that used sera from immunized animals (48, 49). High-resolution proteogenomic-based approaches were developed recently and successfully employed to survey serological antibody repertoires against influenza virus, hepatitis B virus, human cytomegalovirus, norovirus, or SARS-CoV-2 at a monoclonal level, leading to the discovery of neutralizing and/or protective mAb clones that are prevalent in human serum (50–54). Little is known about composition of polyclonal antibody repertoire in EVD survivors. We extended these technical findings by using proteomic serum analysis in combination with single-B-cell paired antibody variable gene sequencing and identified highly-prevalent ebolavirus GP-specific mAbs that are present in convalescent plasma collected from an EVD survivor.

Even though only a small fraction of the peripheral B cell repertoire can be studied, several large-scale studies have revealed an unprecedented genetic diversity of ebolavirus GP-specific memory B cells, with an estimate of many thousands of individual B cell clones circulating in human blood (18, 20). Antibody-mediated protection is, however, limited to several distinct classes of mAbs that recognize vulnerable antigenic sites on the GP and/or can exhibit neutralizing activity and/or Fc-mediated effector functions (21). In the analysis of polyclonal antibody reactivity in convalescent plasma, we identified the preferential recognition of GP_CL_ as a major feature of the secreted antibody profile, as has been also reported previously for four EVD survivors in study by Davis and colleagues (18). These data suggest that the response in which most immunodominant antigenic sites on GP are not fully accessible on the intact GP for recognition by polyclonal antibodies in convalescent plasma is typical. Further, by studying competition-binding with plasma-purified polyclonal antibodies we identified that most of the reactivity to intact GP in plasma is mediated by glycan cap- and base region-specific antibodies, while RBS- and at lesser extent base region-specific antibodies dominated in the response to GP_CL_.

Comprehensive characterization of individual mAbs identified by this proteo-genomic approach confirmed a high serological prevalence of antibodies representing three major classes that we defined based on the antibody binding site - base region, head domain/RBS, or glycan cap. Functional studies revealed that two classes of mAbs, the glycan cap- and base region-specific clones, contributed principally to *in vivo* protection. Given the lower serological prevalence of glycan cap-specific antibodies (as determined by cell-surface-displayed GP binding assays using polyclonal antibodies from plasma), we can speculate that glycan cap-specific antibodies likely contribute to protection to a lesser extent in the polyclonal plasma repertoire than base region-specific antibodies. Notably, we detected one base region-specific mAb that conferred a high level of therapeutic protection *in vivo* and possessed high neutralizing activity (IC_50_ = 6 to 15 ng/mL) comparable or superior to the highly potent mAbs against EBOV that have been described previously (5, 22, 23). This finding suggested the utility of the proteo-genomic approach for the discovery of new potently neutralizing mAbs against ebolaviruses. It should be noted that all mAbs were tested here *in vivo* as recombinant IgG1. The plasma antibody response is more complex because, in addition to neutralizing activity, naturally occurring antibodies in plasma have diverse heavy chain sub-classes, isotypes and allotypes that may play a critical role in protection by mediating diverse Fc-mediated effector function activities (38, 39). A proteomics methodology that would allow coupling of the Fv- and Fc-region information for individual mAbs from serological repertoire is needed.

The overlap between the heavy and light chain variable region sequences of EBOV GP-reactive IgG proteins identified in plasma and of cDNA of antibody genes from EBOV GP-reactive memory B cells was small in this study (48 shared light and 5 shared heavy chain variable region sequences of a total of 1,512 paired antibody heavy and light chain variable region cDNA sequences). This finding is likely due to a much larger diversity for the memory B cell repertoire. Also, a technical limitation of the proteomics approach is such that only the most highly represented proteins are easily detected. Additional shared immunoglobulin clones might be present in the plasma at levels that were not detected. Likely, however, the most physiologically relevant plasma antibodies (those at higher concentration and constituting the bulk of the plasma EBOV GP-reactive repertoire) were identified. It is interesting to note that antibodies from both repertoires recognized the same three major antigenic sites on GP, indicating that this proteo-genomic analysis successfully identified mAbs of each major specificity that were represented in the plasma.

Despite the broad reactivity of polyclonal antibodies in plasma that bound to EBOV, BDBV, and SUDV GPs, most of the neutralizing mAbs identified were specific to the GP of the virus that infected this individual (EBOV). The broadly reactive mAbs we identified bound to cryptic epitopes on GP_CL_. These mAbs were non-neutralizing or weakly neutralizing against virus displaying intact EBOV GP but strongly neutralizing against virus displaying EBOV GP_CL_. Similar features were described for the human MARV neutralizing mAb MR78. This antibody binds to uncleaved MARV GP and EBOV GP_CL_ but not to uncleaved EBOV GP, and it neutralizes only MARV, not EBOV (36). These “MR78-like” antibodies recognizing cryptic epitopes that are shielded by the glycan cap and/or MLD on intact GP appear dominant in plasma. The role for this highly-represented class of plasma antibodies in protective immunity against ebolaviruses remains uncertain. We showed that in the setting of monotherapy, treatment with the mAb EBOV-1133 representing this antibody class did not protect mice against EBOV. However, the reductionist approach of studying such antibodies to cryptic epitopes using monotherapy may not represent the physiologic role for these antibodies in the presence of the full repertoire of GP-reactive antibodies. It is possible that some of the GP-reactive antibodies in the polyclonal repertoire in plasma can facilitate binding of these “non-functional” antibodies to their cryptic epitopes on GP by allosteric effects on the accessibility of occluded sites. Indeed, synergy for binding and neutralization has been shown for several pairs of mAbs with members that recognize the base region or glycan cap on the GP (6, 55). More studies are needed to elucidate role of this class of head domain/RBS-specific antibodies in humoral immunity for ebolaviruses.

A phenomenon of convergence of antibody responses against ebolavirus GP has been described for B cell responses defined by next-generation antibody gene sequencing and isolation of mAb from single GP-reactive B cells. Several recent studies showed that memory B cells in several individuals used genetically similar B cell receptor genes to encode neutralizing mAbs (20, 56, 57). Another study described common structural and genetic features of the glycan cap recognition by several broadly-reactive human mAbs isolated from memory B cells (41). In our study of serological response in one donor we characterized three major epitope classes of mAbs using a relatively small panel of 52 mAbs. Future studies should determine if common molecular features of the polyclonal response that we described for one individual are observed in the plasma response of multiple individuals. At present, this type of study is quite labor-intensive and expensive, but continuing improvement of the efficiency of proteo-genomic methodologies should enable increasingly comprehensive analysis and comparison of antibody repertoires in serum or plasma.

Together, the findings from these combined proteomic and single B cell genetic repertoire studies extend the breadth of current knowledge about the composition and function of the human humoral response to ebolavirus infection and may direct vaccination strategies in the future.

## Data Availability Statement 

All data needed to evaluate the conclusions in the paper are present in the paper or the **Supplementary Material**. Further information and requests for resources and reagents should be directed to and will be fulfilled by the Lead Contact, JC (james.crowe@vumc.org). Materials described in this paper are available for distribution for nonprofit use using templated documents from Association of University Technology Managers “Toolkit MTAs”, available at: https://autm.net/surveys-and742tools/agreements/material-transfer-agreements/mta-toolkit.

## Ethics Statement

The studies involved human participants were approved by the Institutional Review Board of Vanderbilt University Medical Center. Human PBMCs and plasma were collected after written informed consent. The patients/participants provided their written informed consent to participate in this study. The animal protocols for testing of mAbs in mice were approved by the Institutional Animal Care and Use Committee of the University of Texas Medical Branch (UTMB) in compliance with the Animal Welfare Act and other applicable federal statutes and regulations relating to animals and experiments involving animals.

## Author Contributions

PG, AdG, GA, BD, AB, NC, and JC planned the studies. PG, SB, JS, PI, KH, RB, JL, ArG, ED, and BG conducted experiments. PG, AdG, SB, ED, EC, BG, and NC analyzed data and interpreted the studies. EOS provided critical reagents. AB, LZ, and JC obtained funding. PG and JC wrote the first draft of the paper. All authors contributed to the article and approved the submitted version.

## Funding

This work was supported by U.S. N.I.H. grants U19 AI109711 (to JC and AB), 1R43AI129082-01 (to AdG, 1R43AI129082-01 (to LZ), and 1R44GM122102 (to SB and NC), HHS contract HHSN272201400058C (to BD and JC), NIAID U19 109762 (to ES), DTRA grant HDTRA1-13-1-0034 (to JC), and T32 AI138932 (to EC). The project described was supported by the National Center for Research Resources, Grant UL1 RR024975-01, and is now at the National Center for Advancing Translational Sciences, Grant 2 UL1 TR000445-06. This work was supported in part by the “High Resolution and Mass Accuracy Capability” development project at the Environmental Molecular Science Laboratory located at the Pacific Northwest National Laboratory. Work in BSL-4 and ABSL-4 was supported by NIH grant 5UC7AI094660-07 and by the Animal Resource Center of the Galveston National Laboratory. The content is solely the responsibility of the authors and does not necessarily represent the official views of the NIH.

## Conflict of Interest

SB and NC are employees of Abterra Biosciences. JL, ArG, ED, and BD are employees of Integral Molecular. GA is a founder of Seromyx Systems Inc. LZ is employer and co-owner of Mapp Biopharmaceutical. BD is a shareholder of Integral Molecular. AdG was an employee of Mapp Biopharmaceutical. JC has served as a consultant for Luna Biologics, is a member of the Scientific Advisory Board of Meissa Vaccines and is Founder of IDBiologics. The Crowe laboratory at Vanderbilt University Medical Center has received unrelated sponsored research agreements from Takeda Vaccines, IDBiologics and AstraZeneca.

The remaining authors declare that the research was conducted in the absence of any commercial or financial relationships that could be construed as a potential conflict of interest.
