# Supplementary material for: Proteo-Genomic Analysis Identifies Two Major Sites of Vulnerability on Ebolavirus Glycoprotein for Neutralizing Antibodies in Convalescent Human Plasma
Source: Front Immunol. 2021 Jul 16;12:706757. doi: 10.3389/fimmu.2021.706757 (PMC8322977; doi:10.3389/fimmu.2021.706757)
Supplement: Supplementary file 1 [file DataSheet_1.pdf]

Figure S1

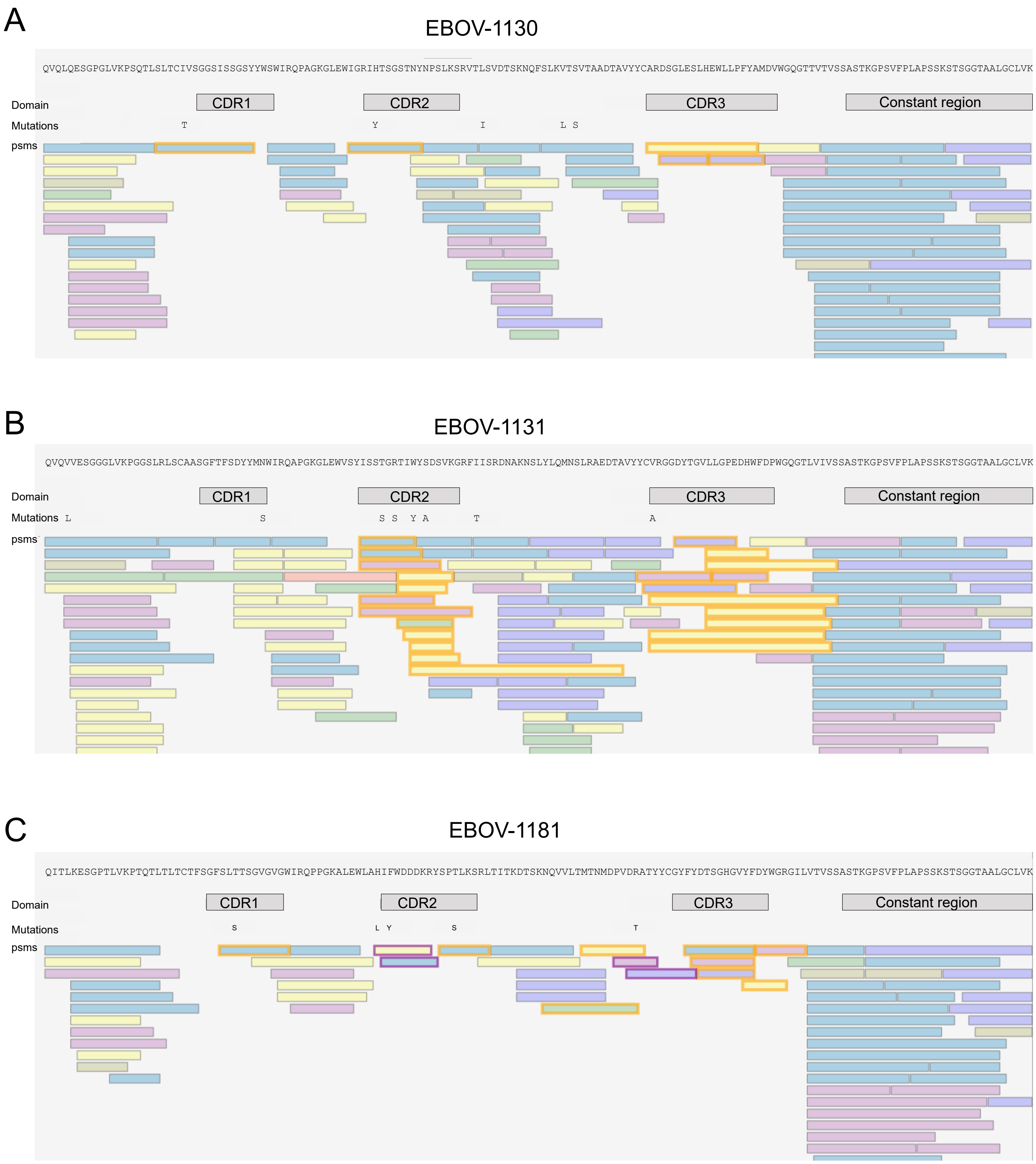
A
EBOV-1130
QVQLQESGPGLVKPSQTLSLTCIVSGGSISSGSYYWSWIRQPAGKGLEWIGRIHTSGSTNYNPSLKSRVTLSVDTSKNQFSLKVTSVTAADTAVYYCARDSGLESLHEWLLPFYAMDVWGQGTTVTVSSASTKGPSVFPLAPSSKSTSGGTAALGCLVK
Domain
CDR1
CDR2
CDR3
Constant region
Mutations
T
Y
I
L S
psms
B
EBOV-1131
QVQVVESGGGLVKPGGSLRLSCAASGFTFSDYYMNWIRQAPGKGLEWVSYISSTGRTIWYSDSVKGRFIISRDNAKNSLYLQMNSLRAEDTAVYYCVRGGDYTGVLLGPEDHWFDPWGQGTLVIVSSASTKGPSVFPLAPSSKSTSGGTAALGCLVK
Domain
CDR1
CDR2
CDR3
Constant region
Mutations
L
S
S S Y A
T
A
psms
C
EBOV-1181
QITLKESGPTLVKPTQTLTLTCTFSGFSLTTSGVGVGWIRQPPGKALEWLAHIFWDDDKRYSPTLKSRLTITKDTSKNQVVLTMTNMDPVDRATYYCGYFYDTSGHGVYFDYWGRGILVTVSSASTKGPSVFPLAPSSKSTSGGTAALGCLVK
Domain
CDR1
CDR2
CDR3
Constant region
Mutations
S
L Y
S
T
psms

Supplementary Figure 1. Identification of mAb sequences in convalescent plasma using proteo-genomics analysis. Peptide coverage maps that were created for each of three memory B cell sequences ([**panel A**] EBOV-1130; [**panel B**] EBOV 1131; [**panel C**] EBOV-1181) by searching the mass spectra against memory B cell sequences dataset are shown. Alicanto analysis software was used to generate maps of peptide coverage; maps of three identified heavy chain variable region sequences are shown as examples. The germline residues for the classified variable gene identifications (defined as "Mutations") and positions of CDR1, CDR2, or CDR3 regions (defined as "Domain") are shown. Each identified spectrum was assigned a peptide sequence defined as peptide-spectrum matches (PSMS), and identical peptides are represented by a single box. The color of each rectangle corresponds to the enzymatic cleavage reaction from which that peptide originated: trypsin (green), chymotrypsin (light blue), pepsin (light pink), elastase (yellow), ArgC (tomato/dark pink), LysC (olive), or GluC (dark red). Gold outline of the rectangle indicates the peptide maps that are unique to that single antibody variable region sequence. The peptide maps unique to that antibody CDR1-CDR3 clone (*i.e.*, concatenating CDR1+CDR3 and uniquely clustering on those regions define the CDR1-CDR3 clone) are shown with purple outline.

Supplementary Table 2. Peptides for CDR3 of heavy and light chain variable regions identified by proteo-genomic approach

	Rank*	CDR3	# peptides
**Heavy chain**	1	VRGGDYTGVLLGPEDHWFDP	15
	2	GYFYDTSGHGVYFDY	5
	3	ARDSGLESLHEWLLPFYAMDV	3
	4	ARDPTYSSGWRDY	2
	5	AKGGIWF	1
**Light chain**	1	QQYGSSPRT	10
	2	QQAKSFPLT	9
	3	QQAKSYPIT	8
	4	MQALQTPLT	8
	5	QQYYSTPRT	7
	6	QQRSNWPFT	7
	7	QQYNSYPFT	6
	8	QQAKSFPIT	6
	9	MQALQTPIT	6
	10	QQYGSSPLT	6
	11	QQYDSSPWT	5
	12	MQGRETPRT	5
	13	MQALQTPRT	5
	14	QQANSFPQT	5
	15	QQRSRWPLT	4
	16	QKYNSAPFT	4
	17	QQYDNLPLT	4
	18	QQYNNWLPWT	4
	19	QVWHSIGDQQV	4
	20	QKYNSAPWT	3
	21	QQYNNWPPTWT	3
	22	QQYYSTPWT	3
	23	QKYNSAPLT	3
	24	QQRDKWPLT	3
	25	QQYNSYPWT	3
	26	QQYYSAPRT	3
	27	QYTNSFPLT	3
	28	QQYYSIPWT	3
	29	QQRTSWPPLT	3
	30	QQYDNVPLT	3
	31	QQYNNWPRT	3
	32	QQRSNWPHT	2
	33	QQRTNRPPLFT	2
	34	QSADRSGTYVV	2
	35	QQSYSTPIT	2
	36	QQRSNWPPCT	2
	37	QQYDSWPRT	2
	38	QKYDSAPWT	2
	39	QQSYSTLCT	1
	40	QQRASWPPEIT	1
	41	QQYYSIP	1
	42	QQTYSTPTWT	1
	43	QKYNSAPLT	1
	44	QQRSNRFT	1
	45	QQYGNSPVT	1
	46	QQYNSYSQT	1
	47	QQSYTRRT	1
	48	QEYKTWT	1

*Ranking is based on identified peptide count for peptides covering the CDR3 region of heavy and light chain variable regions when searched against memory B cell paired repertoire dataset

Supplementary Table 3. Inferred antibody germline genes and variable region analysis of represented in plasma EBOV GP-reactive antibodies

Antibody number (EBOV-*x*)	V-gene (*IGHV*)	D-gene (*IGHD*)	J-gene (*IGHJ*)	Isotype* (*IGH*)	HCDR3	LCDR3
1130	*4-61*02*	*3-3*01*	*6*02*	M	ARDSGLESLHEWLLPFYAMDV	QQSHSSPYT
1131	*3-11*01*	*2-8*02*	*5*02*	G1	VRGGDYTGVLLGPEDHWFDP	QQAYRTPYT
1132	*1-2*02*	*6-19*01*	*4*02*	G1	ARDPTYSSGWRDY	QQYYSMPIT
1133	*3-11*01*	*3-9*01*	*4*02*	G1	AKGGIWF	HQYGRSPFT
1134	*3-30*02*	*2-2*02*	*4*02*	M	AKFYSQYCSSTSCYILDY	QQRSNRFT
1135	*3-15*01*	*1-14*01*	*5*02*	G1	STGPGSLTDLATGTPFDL	QQRTNRPPLFT
1136	*3-48*01*	*3-10*01*	*4*02*	G1	TREDAVTLVRGVVLGPHYFDS	QQSYTRRT
1137	*4-61*02*	*2-2*01*	*4*02*	M	AGFHCSSGACKAEDY	QQYYSAPRT
1138	*3-13*01*	*6-6*01*	*3*02*	G1	ARAVHSSSAIFDI	QQYGSSPLT
1139	*5-51*03*	*3-22*01*	*6*02*	G1	ARHPPHAGSYYYHGMDV	QQRSNWPHT
1140	*5-51*03*	*3-16*01*	*6*02*	D	ARHGGGGTGGSVYYYYGMDV	QQYNNWPPTWT
1141	*4-34*01*	*3-3*01*	*4*02*	G3	ARARDYDFWTGYSRFFDH	QQRSNWPPCT
1142	*1-69*11*	*3-3*01*	*5*02*	G1	VRDPISRNWLDP	QQYDNVPLT
1143	*4-59*01*	*5-18*01*	*6*03*	M	ARGGYSYGFSPYYYYMDV	QKYNSAPFT
1144	*4-34*01*	*5-18*01*	*6*03*	G2	TRGRRIPFHYRGFSYGYEYYYYMDV	MQGRETPRT
1145	*3-73*01*	*1-14*01*	*4*02*	G1	TSTGEEFDY	QKYNSAPWT
1146	*4-61*02*	*2-2*01*	*6*02*	D	AREPVGYCSSTSCYPYYYYYYGMDV	MQALQTPRT
1147	*3-53*01*	*3-3*01*	*6*02*	A1	ARIYYYYYGMDV	QKYDSAPWT
1148	*3-48*03*	*5-18*01*	*4*02*	A1	ARDTTWIRLDY	QQRASWPPEIT
1149	*3-21*02*	*3-9*01*	*6*03*	G1	ARVPFPDLLTGDYLLGYYYMDV	QQRDKWPLT
1150	*4-39*07*	*3-10*01*	*4*02*	M	ANPWFGEANDDY	QQRSRWPLT
1151	*3-30*02*	*6-6*01*	*6*02*	M	AKVGERNSSSSIRWGYYYYGMDV	QQANSFPQT
1152	*3-30*02*	*3-22*01*	*3*02*	M	ASLGYYYDSSGYYYGPNLAFDI	QKYNSAPLT
1153	*4-59*01*	*6-25*01*	*3*02*	G1	AREDSSGYFMNAFDI	QQYDSWPRT
1154	*1-2*02*	*3-3*01*	*3*02*	G3	ARAATTFGVVLSRANDAFDI	QVWHSIGDQQV
1155	*3-53*02*	*1-14*01*	*3*02*	G1	ARGSGWNHVPGAFDI	QQYNSYPWT
1156	*3-11*01*	*6-6*01*	*6*02*	M	ARDKWDGVAARPHYYYGMDV	QQSYSTLCT
1157	*4-38-2*02*	*2-2*01*	*4*02*	G1	ARDSPLVVVSAANSGLFDS	QQYYSTPWT
1158	*1-69*08*	*2-21*02*	*4*02*	G1	ARDLGTPVTEKDDY	QQYNSYPFT
1159	*3-48*03*	*3-22*01*	*4*02*	M	ARARDPRTGSSGYLRY	QQYDNLPLT
1160	*3-11*04*	*1-20*01*	*6*03*	G1	ARVNGYNYGPLTGYHYMDV	QQTYSTPTWT
1161	*3-11*04*	*3-22*01*	*4*02*	G1	ASTTWLYDSSGYYPDY	QQRSNWPFT
1162	*3-7*01*	*1-26*01*	*6*04*	G1	ARVPDHEGAKWDLLWPTYYHYMDV	QSADRSGTYVV
1163	*3-66*02*	*6-19*01*	*1*01*	G1	ARLLGSGPVFMEYFQH	QQAKSFPLT
1164	*1-2*02*	*2-15*01*	*4*02*	G1	ARDPTYISGGWRDY	QQYYSIPIT
1165	*4-34*01*	*7-27*01*	*6*03*	G2	ARRIQRGQTYWGFMDV	MQALQTPIT
1166	*3-15*01*	*3-9*01*	*4*02*	G1	TTDTLRDNYDDILTGYYRGFDY	QQRTSWPPLT
1167	*4-59*01*	*2-2*01*	*2*01*	M	TSSSSHYFYFDF	QQYNNWLPWT
1168	*3-11*01*	*6-13*01*	*4*02*	M	ARDRGSSWYQMGIFDY	QQYYSTPRT
1169	*2-5*02*	*2-8*01*	*4*02*	G1	GYFYDTRGYGVYFDY	QQAKSFPIT
1170	*1-69*14*	*1-26*01*	*6*03*	M	ARTLFAGSYYYYYYMDV	QQYNSYSQT
1171	*3-21*01*	*6-6*01*	*4*02*	G2	ARGGSIAARSPFDY	QEYKTWT
1172	*1-69*11*	*6-13*01*	*4*02*	G1	ARDVIGSHSGSSWAYYYFDY	QQYGSSPRT
1173	*1-18*01*	*4-17*01*	*6*03*	G2	ARLSHDFGDLGSLFYYFYMDV	QYTNSFPLT
1174	*3-49*04*	*6-6*01*	*6*02*	G1	ARVGTYSSSSHIVYYYYGMDV	QQYDSSPWT
1175	*2-5*02*	*2 -8*02*	*4*02*	G1	GYFYDTRGHGVYFDY	QQAKSYPIT
1176	*3-21*01*	*6-13*01*	*3*01*	G1	ARDGPRTVPAAGIGDAFHV	QQYYSIPWT
1177	*1-69*08*	*3-22*01*	*3*02*	G2	ARPTEYHDSSGYLATHNLEAFDI	QQYGNSPVT
1178	*3-30*02*	*1-26*01*	*4*02*	D	AKDRGSYRYFDY	QQYNNWPRT
1179	*3-74*01*	*1-26*01*	*6*02*	D	ARVSREHPYGMDV	QQSYSTPIT
1180	*3-21*01*	*3-9*01*	*4*02*	G1	ARDSGRGGYYKGTDY	MQALQTPLT
1181	*2-5*02*	*3-22*01*	*4*02*	G1	GYFYDTSGHGVYFDY	QQAKSYPIT

* Heavy chain isotype identified from paired memory B cell antibody variable gene sequencing analysis

Supplementary Table 4. Binding and functional properties of individual mAbs determined by micro-scale screening assays

Antibody number (EBOV-*x*)	Micro-scale purified antibody concentration (μg/mL)*	GP binding by ELISA (optical density at 450 nm)**			Binding to Jurkat cell-surface displayed EBOV GP (log_{10} MFI)**		EBOV neutralization (%)***	Antibody-mediated cellular phagocytosis (score)***
		EBOV GP	BDBV GP	SUDV GP	Uncleaved	+ thermolysin		
1130	408	3.5	3.5	0.1	3.9	5.6	73	95
1131	36	3.6	3.3	0.2	4.2	4.8	10	101
1132	48	3.6	3.0	1.0	6.2	4.2	20	110
1133	60	3.6	3.6	3.6	3.9	6.3	16	102
1134	84	3.5	3.5	2.6	3.7	3.7	9	106
1135	600	3.4	2.4	3.2	4.0	3.8	17	108
1136	60	3.6	0.7	0.2	4.9	5.5	51	97
1137	72	2.9	0.9	0.3	4.1	3.9	31	101
1138	84	3.5	1.8	0.3	3.8	6.0	1	123
1139	96	3.5	3.5	1.9	4.5	6.2	-9	134
1140	24	1.4	0.8	0.3	4.3	3.8	-7	153
1141	24	2.3	2.1	0.7	5.1	4.8	-7	153
1142	108	1.8	1.3	0.9	3.7	3.8	-10	121
1143	36	1.5	1.0	0.3	3.6	3.6	-6	109
1144	48	2.6	0.8	0.1	5.7	6.4	44	136
1145	18	2.0	0.6	0.1	4.1	3.8	17	142
1146	168	2.5	1.5	0.7	3.6	3.6	-1	77
1147	60	3.5	3.4	3.4	3.7	4.4	-9	91
1148	36	2.9	2.9	0.1	3.9	3.9	-9	167
1149	108	3.7	3.6	0.2	5.2	5.5	11	164
1150	48	2.2	1.4	0.7	3.6	3.8	-10	171
1151	24	3.4	2.1	0.3	3.6	3.9	-10	140
1152	36	3.6	3.5	0.1	4.6	3.9	1	140
1153	30	3.6	3.6	0.2	4.6	5.1	12	121
1154	36	3.5	3.5	0.1	3.7	4.0	7	103
1155	36	0.1	0.1	0.1	3.6	3.7	-8	122
1156	72	0.3	0.1	0.1	3.8	3.6	-10	53
1157	12	3.5	1.3	0.4	4.3	3.7	-9	110
1158	36	3.3	2.7	0.1	4.0	4.0	-11	99
1159	72	1.9	1.0	0.1	3.6	3.6	-14	62
1160	36	3.6	3.5	0.1	5.1	5.6	-4	144
1161	42	3.6	3.6	0.2	4.9	3.9	14	148
1162	48	2.8	0.3	0.1	4.9	5.5	3	92
1163	36	3.6	3.5	0.1	5.3	5.6	0	154
1164	72	3.6	0.1	0.2	6.1	4.2	80	165
1165	168	0.4	0.1	0.1	4.5	3.9	-12	174
1166	120	3.6	3.5	0.1	5.6	5.5	10	159
1167	24	2.2	1.8	0.1	3.6	3.7	-12	195
1168	2	1.1	0.5	0.1	3.7	4.0	-10	145
1169	198	3.6	3.2	0.1	5.9	5.5	29	126
1170	24	0.4	0.1	0.1	3.9	3.9	17	67
1171	24	2.8	1.3	0.2	3.6	4.0	96	162
1172	72	3.6	3.6	2.8	5.1	5.8	-12	101
1173	24	3.1	3.6	0.6	3.7	5.2	-12	168
1174	12	3.5	3.5	0.1	4.7	5.4	-10	194
1175	36	3.6	3.1	0.1	4.9	4.7	45	191
1176	60	3.6	1.0	0.1	5.1	5.9	98	169
1177	96	3.6	3.6	2.4	5.3	5.6	96	159
1178	36	0.4	0.1	0.1	4.0	3.9	6	64
1179	60	3.2	0.1	0.1	4.1	4.1	10	118
1180	60	3.6	0.1	0.1	5.6	6.3	20	137
1181	48	3.6	0.1	0.1	5.8	5.4	28	159

**Controls**								
EBOV-520	43	3.6	3.6	2.1	4.6	6.4	49	172
13C6 (5 μg/mL)	N/A	N/A	N/A	N/A	5	3.6	N/A	186
CO5 (Flu HA)	66	0.2	0.1	0.1	3.7	4.0	-10	63

* each purified mAb had a total volume of 100 μL

** each mAb was tested at a single 1:10 dilution

*** each mAb was tested at a single 1:6 dilution
